# Latent profiles of day-7 cognitive performance and associated ecological factors in older adults after elective surgery: an observational study

**DOI:** 10.3389/fpsyt.2026.1975739

**Published:** 2026-10-28

**Authors:** Yao Liao, Xuanying Zhang, Yunlei Zhang, Lunmei Shi, Yalin Liu

**Affiliations:** 1 Department of Cardiothoracic and Vascular Surgery, Jingzhou Central Hospital, Jingzhou, China; 2 Medical School, Yangtze University, Jingzhou, China; 3 Department of Neurology, Jingzhou Central Hospital, Jingzhou, China

**Keywords:** cognitive performance, elective surgery, health ecological model, latent profile analysis, older adults, perioperative Nursing

## Abstract

**Objective:**

To identify latent profiles of postoperative day-7 cognitive performance among older adults after elective surgery and to explore demographic, behavioral, clinical, and social factors associated with profile membership.

**Methods:**

This prospective observational study included 308 consecutively recruited patients aged ≥65 years who underwent elective surgery under general anesthesia. Cognition was assessed with the Mini-Mental State Examination (MMSE), and latent profile analysis used five study-defined postoperative domain scores. Candidate correlates were organized with the health ecological model and examined using univariate tests and posterior-probability-weighted exploratory multinomial regression.

**Results:**

Three severity-ordered profiles were identified: low (n=83, 26.9%), intermediate (n=72, 23.4%), and high postoperative cognitive performance (n=153, 49.7%). Older age and lower education were associated with both lower-performance profiles. Greater activities-of-daily-living dependence, lower income, and living alone were associated with the low-performance profile, whereas poorer sleep was associated with the intermediate-performance profile.

**Conclusions:**

Day-7 MMSE performance formed a severity gradient across three data-driven profiles. These cross-sectional profiles do not demonstrate postoperative decline, a clinical diagnosis, or an effect attributable specifically to general anesthesia. Longitudinal studies with retained baseline scores, appropriate comparison groups, and broader neuropsychological assessment are required before profile-specific interventions can be recommended.

## 1 Introduction

Cognitive performance after surgery is clinically important in older adults because reduced physiological and cognitive reserve may increase vulnerability during recovery (1). Postoperative scores can reflect preexisting cognitive reserve, education, perioperative stressors, illness, medications, sleep, pain, and measurement effects. A recent meta-analysis reported that postoperative neurocognitive outcomes are common after non-cardiac surgery, although valid diagnosis of postoperative cognitive change requires comparison with baseline and appropriate diagnostic criteria rather than a single postoperative screening score (2). Poor cognitive performance may complicate recovery and compromise independence (3).

Potential correlates are diverse. Advanced age and lower education have been associated with poorer perioperative cognitive outcomes (3), while recent work implicates cognitive reserve, frailty, social conditions, and sleep (4, 5). Functional dependence may reflect diminished systemic reserve; low income can restrict access to nutrition, rehabilitation, and follow-up; and living alone can reduce cognitive stimulation and practical support. Longitudinal evidence also links social isolation with poorer late-life cognition (6). A multilevel framework may therefore help organize the biological, behavioral, functional, and socioeconomic conditions associated with postoperative cognitive performance.

Postoperative cognitive performance may vary substantially in severity across older surgical patients. A single MMSE total score can obscure the relative pattern of domain scores, while latent profile analysis (LPA) can identify data-driven subgroups from multiple continuous indicators. However, parallel class trajectories may represent categories along one general cognitive-performance dimension rather than qualitatively distinct phenotypes. Accordingly, profile labels must describe observed postoperative levels without implying a diagnosis or longitudinal decline that was not modeled.

The health ecological model provides a multilevel framework for organizing individual biological characteristics, behaviors and functional conditions, and social context (7, 8). In this study, it was used *a priori* as an interpretive organizing framework for candidate correlates. We did not test hierarchical block effects, mediation, interactions, or causal pathways; ecological mechanisms are therefore interpreted cautiously.

This study aimed to identify latent profiles of postoperative day-7 cognitive performance among older adults after elective surgery and to examine factors associated with profile membership. All participants received general anesthesia, but the design was not intended to estimate an effect of anesthesia because it included neither a regional-anesthesia nor a non-surgical comparison group.

## 2 Materials and methods

### 2.1 Study design and setting

We conducted a prospective observational study with a cross-sectional LPA of the postoperative day-7 assessment. Participants were consecutively recruited from older patients undergoing elective surgery under general anesthesia at a tertiary hospital in Jingzhou, Hubei Province, China, between May and July 2026. The study was reported with reference to the Strengthening the Reporting of Observational Studies in Epidemiology (STROBE) statement (9).

### 2.2 Participants and sampling

We used consecutive sampling to recruit all eligible patients in the order in which they underwent elective surgery during the study period.

#### 2.2.1 Inclusion criteria

The inclusion criteria were: (1) age ≥65 years; (2) scheduled elective surgery under general anesthesia; (3) preoperative MMSE score >24 according to the prespecified eligibility threshold; (4) ability to communicate and complete the questionnaires; and (5) written informed consent. The final analytic sample ranged from 65 to 96 years of age.

#### 2.2.2 Exclusion criteria

The exclusion criteria were: (1) a documented preoperative diagnosis of dementia, mild cognitive impairment, delirium, or another major neurocognitive disorder; (2) a severe psychiatric disorder that prevented valid assessment; (3) severe visual, hearing, or communication impairment; (4) perioperative medication use that, in the investigators’ judgment, precluded valid cognitive assessment; and (5) incomplete key study data. Preexisting mild cognitive impairment and psychiatric diagnoses were ascertained by preoperative medical-record review; the MMSE cutoff was an eligibility screen and was not treated as a diagnostic test.

### 2.3 Sample size calculation

The recruitment target was informed by an expected postoperative cognitive-outcome prevalence of approximately 23% on postoperative day 7 (2), 16 candidate variables, and the anticipated need to estimate several latent profiles. A conventional exploratory rule of 10–15 observations per candidate variable produced a target of 240 participants; allowing for approximately 20% incomplete data yielded a target of 300. This was an approximate recruitment target rather than a formal power calculation for LPA or multinomial regression. Because the smallest retained class contained 72 participants, regression precision and sparse-cell estimates were considered explicitly when interpreting the findings.

### 2.4 Measures

The following instruments and variables were used to operationalize the health ecology framework and assess postoperative cognitive and clinical status.

#### 2.4.1 Health ecology framework

The health ecological model was used only as an interpretive organizing framework. Downstream factors comprised age, sex, obesity, multimorbidity (at least two chronic conditions), and nutritional risk assessed with Nutritional Risk Screening 2002. Midstream factors comprised smoking, alcohol use, sleep quality, activities of daily living (ADL), and pain. Upstream factors comprised education, employment, residence, living arrangement, and monthly per-capita household income. No mediation, interaction, causal pathway, or blockwise ecological effect was tested.

#### 2.4.2 General information questionnaire

A researcher-developed questionnaire captured prespecified variables. Trained researchers conducted structured face-to-face interviews and verified clinical variables from hospital records. Smoking and alcohol use were recorded as current self-reported use (yes/no) at enrollment. Employment was recorded as employed or not employed; residence as urban, town, or rural; living arrangement as living alone or not living alone; and monthly per-capita household income as <CNY1,000, CNY1,000–2,999, CNY3,000–4,999, or ≥CNY5,000. Body mass index was calculated as kg/m² and categorized as underweight (<18.5), normal (18.5 to <24), overweight (24 to <28), or obese (≥28). Multimorbidity was defined *a priori* as two or more chronic conditions.

#### 2.4.3 Mini-mental state examination

Trained research assistants administered the Chinese Mini-Mental State Examination (MMSE), a 30-point screening instrument (10–12). For this study, items were grouped into five prespecified domains: orientation (10 points), immediate registration/memory (3 points), attention and calculation (5 points), delayed recall (3 points), and language and visuoconstruction (9 points). We replaced the earlier term ‘language and executive function’ because the standard MMSE does not contain a dedicated executive-function domain. The preoperative assessment was used only to confirm the eligibility threshold (>24); individual continuous baseline scores were not retained in the final analytic dataset and could not be used to estimate change or reliable change. The same standard MMSE format was used at postoperative day 7; no MMSE-2 alternate form or formal practice-effect correction was applied. The five day-7 domain scores were the LPA indicators, and profile labels denote relative postoperative performance rather than clinical diagnoses or change from baseline.

#### 2.4.4 Barthel index

Functional independence was assessed on postoperative day 7 with the 10-item Barthel Index (13). Scores range from 0 to 100, with higher scores indicating greater independence; 100 was classified as independent, 61–99 as mild dependence, 41–60 as moderate dependence, and ≤40 as severe dependence. The instrument covers feeding, bathing, grooming, dressing, continence, toilet use, transfers, mobility, and stairs. Reliability studies have reported close agreement across assessment sources and internal consistency of approximately 0.87–0.90 (14, 15). Trained researchers rated actual performance and checked uncertain responses against nursing records or caregiver information.

#### 2.4.5 Visual analog scale for pain

Pain intensity was assessed on postoperative days 3 and 7 with a 10-cm visual analog scale (VAS) anchored by ‘no pain’ and ‘worst imaginable pain’ (16). Scores were categorized as no pain (0), mild (1–3), moderate (4–6), or severe (7–10). The VAS has demonstrated acceptable psychometric performance in older adults, although completion errors can occur with cognitive or psychomotor impairment (17); researchers therefore confirmed understanding before recording the score.

#### 2.4.6 Nutrition Risk Screening 2002

Nutritional risk was assessed at enrollment with Nutritional Risk Screening 2002 (NRS-2002), which combines impaired nutritional status (0–3), disease severity (0–3), and one additional point for age ≥70 years; a total score ≥3 indicates risk (18, 19). Validation work reported 80.3% sensitivity, 79.8% specificity, and inter-rater κ=0.72 against reference nutritional assessments (20). Trained researchers completed the screen from interview and clinical-record data.

#### 2.4.7 Richards-Campbell Sleep Questionnaire

Sleep was assessed on postoperative day 7 with the Chinese Richards–Campbell Sleep Questionnaire (RCSQ-C). Its five visual analog items assess sleep depth, latency, awakenings, return to sleep, and overall quality; their mean yields a 0–100 score, with higher scores indicating better sleep. The original instrument showed Cronbach’s α=0.90 (21), and the Chinese version showed α=0.923, corrected item–total correlations of 0.680–0.805, and a one-factor solution explaining 76.6% of variance (22). Prespecified categories were very good (76–100), good (51–75), poor (27–50), and very poor (<26). Regression coding increased from very good to very poor.

### 2.5 Data collection and quality control

Two trained research assistants assessed eligibility, obtained written consent, and collected questionnaire and record-based data. Preoperative medical-record review was used to ascertain documented mild cognitive impairment, dementia, delirium, and severe psychiatric disorders. The preoperative MMSE was used for eligibility only; its continuous total and domain scores were not retained in the final analytic dataset. Postoperative MMSE, Barthel Index, sleep, and day-7 pain assessments were completed on postoperative day 7 in a quiet setting, while an additional pain assessment was completed on day 3. Two assistants independently entered data into EpiData 3.1 and resolved discrepancies against source documents.

### 2.6 Statistical analysis

Analyses used two-sided α=0.05. Categorical variables are reported as n (%). Continuous and ordered variables are reported as median (P25, P75) and compared with the Kruskal–Wallis H test; nominal variables were compared using Pearson’s χ² test. Degrees of freedom and effect sizes are reported for key comparisons. Sparse cells were interpreted cautiously.

Gaussian LPA models with one through four profiles were fitted to the five raw postoperative MMSE domain scores. Class-specific means were estimated; residual variances were constrained equal across classes and residual covariances were fixed at zero. Models used maximum likelihood, 1,000 random starts, and no imputation because all five indicators were complete. For the selected three-profile solution, the best log-likelihood was reproduced in 997 starts. Selection considered AIC, BIC, sample-size-adjusted BIC, entropy, the Lo–Mendell–Rubin adjusted likelihood-ratio test, bootstrap likelihood-ratio test, class size, parsimony, and interpretability. Average posterior assignment probabilities were calculated. Because the indicators have different ranges, an affine standardized-indicator sensitivity analysis was performed and yielded the same modal classification. Spearman domain correlations, standardized class means, and principal-component analysis were examined to evaluate whether profiles reflected distinct shapes or a general severity dimension.

Exploratory multinomial regression used the high-performance profile as reference. Six variables that met the prespecified univariate screening threshold and were not LPA indicators—age, education, ADL category, income, living arrangement, and sleep—were entered simultaneously. Age was continuous; education, ADL, income, and sleep were coded as ordered one-category increments; living alone was coded 1. To incorporate classification uncertainty, posterior probabilities were used as fractional outcomes in a sensitivity model; estimates were nearly identical to modal-assignment estimates, and posterior-weighted results are reported in **Table 1**. Because univariate screening, ordinal linearity assumptions, the smallest class, and sparse living-arrangement cells may cause instability, all associations are considered exploratory rather than predictive.

**Table 1:** Multinomial logistic regression of factors associated with latent postoperative cognitive profiles.

Variable	Low postoperative cognitive performance vs high postoperative cognitive performance	Low postoperative cognitive performance vs high postoperative cognitive performance	Low postoperative cognitive performance vs high postoperative cognitive performance	Low postoperative cognitive performance vs high postoperative cognitive performance	Intermediate postoperative cognitive performance vs high postoperative cognitive performance	Intermediate postoperative cognitive performance vs high postoperative cognitive performance	Intermediate postoperative cognitive performance vs high postoperative cognitive performance	Intermediate postoperative cognitive performance vs high postoperative cognitive performance
	β	OR	95% CI	P value	β	OR	95% CI	P value
Intercept	-7.961	–	–	0.009	-7.683	–	–	0.003
Age	0.135	1.145	1.053–1.245	0.002	0.106	1.111	1.034–1.194	0.004
Education	-1.256	0.285	0.181–0.447	<0.001	-0.475	0.622	0.446–0.868	0.005
ADL category	0.676	1.966	1.130–3.420	0.017	-0.014	0.986	0.600–1.622	0.957
Monthly per-capita income	-0.548	0.578	0.364–0.919	0.020	0.026	1.026	0.681–1.545	0.902
Living arrangement	3.490	32.780	11.496–93.472	<0.001	0.133	1.142	0.290–4.500	0.850
Sleep quality	0.384	1.468	0.896–2.406	0.127	0.459	1.583	1.057–2.371	0.026

The high postoperative cognitive performance profile was the reference. Posterior class probabilities were used as fractional outcomes to incorporate classification uncertainty. ORs for age represent one year; ORs for education, ADL, income, and sleep represent one-category increases. Modal-assignment estimates were materially unchanged. The living-alone estimate remains vulnerable to sparse-data inflation.

### 2.7 Ethics

The study was approved by the Ethics Committee of Jingzhou Central Hospital (approval no. 2026-285-01). All participants provided written informed consent. Data were de-identified and used only for research purposes.

## 3 Results

### 3.1 Participant characteristics

Data were collected from 312 patients; four records with incomplete key data were excluded, leaving 308 participants. The median age was 68 years (P25, P75: 66, 70; range 65–96), and 164 (53.2%) were men. Education was distributed as follows: illiterate, 16 (5.2%); primary school, 120 (39.0%); junior middle school, 97 (31.5%); senior high/vocational school, 61 (19.8%); and college or above, 14 (4.5%). Forty-eight (15.6%) currently smoked and 60 (19.5%) currently used alcohol. Eighteen (5.8%) were underweight, 167 (54.2%) had normal weight, 100 (32.5%) were overweight, and 23 (7.5%) were obese.

### 3.2 Selection and characteristics of latent profiles

Fit indices improved markedly from one to three profiles (**Table 2**). The three-profile model had entropy=0.963; the Lo–Mendell–Rubin and bootstrap likelihood-ratio tests favored three over two profiles (both P<0.001). Although the four-profile model had lower information criteria, its Lo–Mendell–Rubin test was not significant (P = 0.825), one class contained only 5.5% of participants, and its best solution was reproduced less consistently. We therefore retained the more parsimonious three-profile model. Average posterior probabilities were 1.000, 0.978, and 0.977 for the low-, intermediate-, and high-performance profiles, respectively.

**Table 2:** Fit indices for latent profile models of day-7 postoperative cognitive performance in older adults.

Number of profiles	AIC	BIC	aBIC	Entropy	LMR P value	BLRT P value	Class proportions
1	4815.948	4853.249	4821.533	–	–	–	–
2	3237.335	3297.016	3246.271	1.000	<0.001	<0.001	0.26948/0.73052
3	2946.878	3028.940	2959.166	0.963	<0.001	<0.001	0.26948/0.24015/0.49037
4	2771.519	2875.962	2787.157	0.998	0.825	<0.001	0.05519/0.21644/0.28356/0.44481

AIC, Akaike information criterion; BIC, Bayesian information criterion; aBIC, sample-size-adjusted Bayesian information criterion; LMR, Lo–Mendell–Rubin adjusted likelihood-ratio test; BLRT, bootstrap likelihood-ratio test. Class proportions are model-estimated and ordered from the lowest to the highest model-estimated total cognitive score. Modal counts for the selected three-profile solution were 83, 72, and 153.

The low-performance profile included 83 participants (26.9%), the intermediate-performance profile 72 (23.4%), and the high-performance profile 153 (49.7%). Model-estimated domain means for the low, intermediate, and high profiles were, respectively: orientation, 6.518, 8.389, and 9.432; immediate registration/memory, 1.145, 2.088, and 2.844; attention and calculation, 2.422, 3.609, and 4.715; delayed recall, 0.819, 2.016, and 2.853; and language and visuoconstruction, 4.988, 8.478, and 8.587 (**Figure 1**). Standardized observed means and 95% confidence intervals are reported in **Supplementary Table S1**.

**Figure 1:**
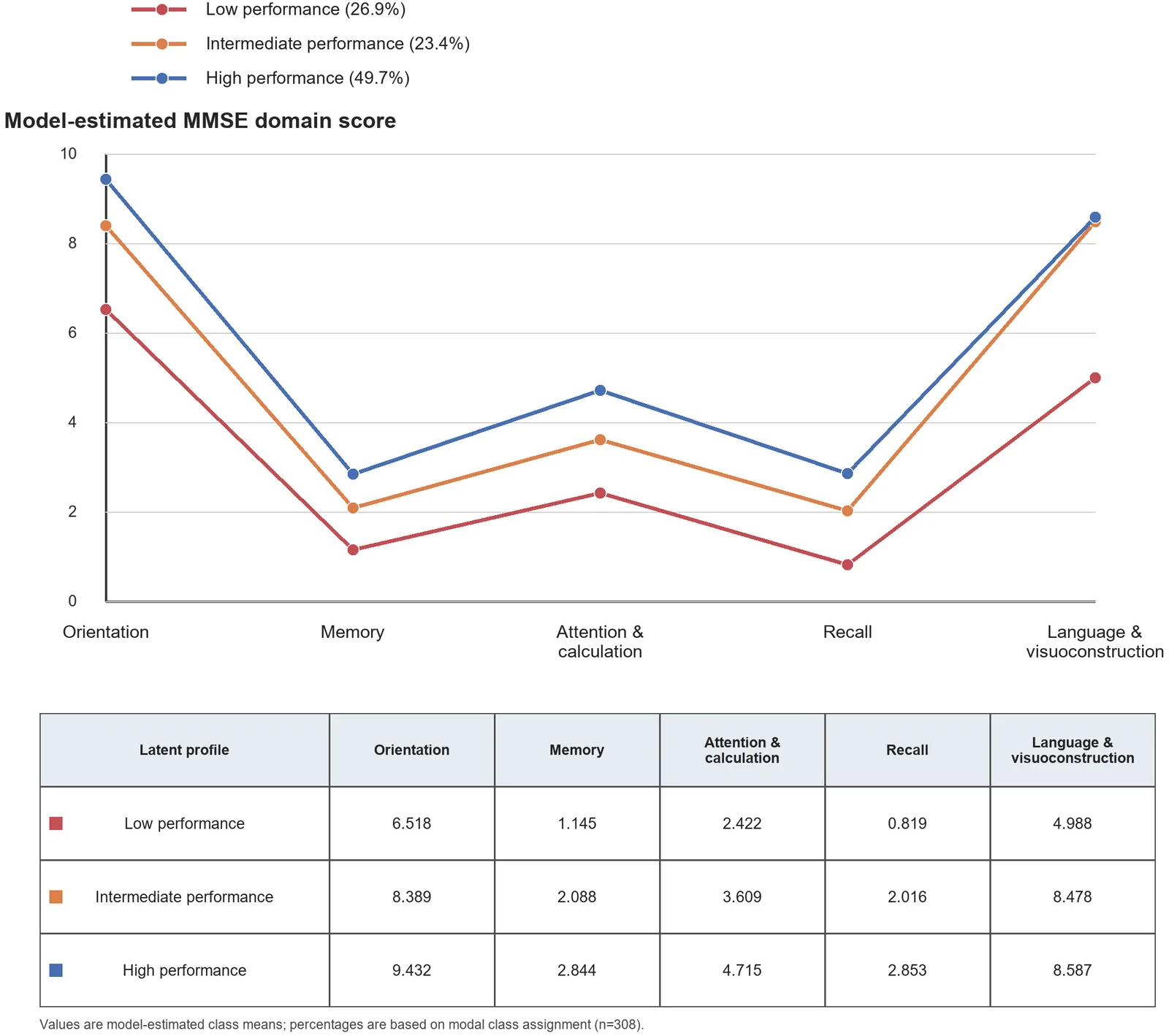
Model-estimated MMSE domain score.

### 3.3 Univariate differences between profiles

The profiles differed by age (H = 17.110, df=2, P<0.001; ϵ²=0.050), education (H = 50.460, df=2, P<0.001; ϵ²=0.159), ADL category (H = 9.345, df=2, P = 0.009; ϵ²=0.024), income (χ²=86.337, df=6, P<0.001; Cramér’s V = 0.374), living arrangement (χ²=79.066, df=2, P<0.001; Cramér’s V = 0.507), and sleep quality (H = 12.722, df=2, P = 0.002; ϵ²=0.035) (**Table 3**). In the low-performance profile, 73.5% had primary education or less, 47.0% lived alone, and 43.4% had monthly per-capita income <CNY1,000. Corresponding living-alone proportions were 4.2% and 5.2% in the intermediate- and high-performance profiles. Other measured characteristics did not differ significantly across profiles.

**Table 3:** Univariate analysis of demographic and clinical characteristics across latent postoperative cognitive profiles.

Variable	Category/statistic	Overall (n=308)	Low postoperative cognitive performance (n=83)	Intermediate postoperative cognitive performance (n=72)	High postoperative cognitive performance (n=153)	Statistic	P value	Test
Sex	Male	164 (53.2)	37 (44.6)	37 (51.4)	90 (58.8)	4.517	0.105	chi-square
	Female	144 (46.8)	46 (55.4)	35 (48.6)	63 (41.2)			
Age, years	Median (P25, P75)	68.00 (66.00, 70.00)	69.00 (67.00, 72.00)	69.00 (66.00, 72.00)	67.00 (65.00, 69.00)	17.110	<0.001	H
BMI category	Underweight	18 (5.8)	7 (8.4)	3 (4.2)	8 (5.2)	3.810	0.702	chi-square
	Normal weight	167 (54.2)	42 (50.6)	45 (62.5)	80 (52.3)			
	Overweight	100 (32.5)	27 (32.5)	20 (27.8)	53 (34.6)			
	Obese	23 (7.5)	7 (8.4)	4 (5.6)	12 (7.8)			
Education	Illiterate	16 (5.2)	13 (15.7)	1 (1.4)	2 (1.3)	50.460	<0.001	H
	Primary school	120 (39.0)	48 (57.8)	33 (45.8)	39 (25.5)			
	Junior middle school	97 (31.5)	13 (15.7)	26 (36.1)	58 (37.9)			
	Senior high/vocational school	61 (19.8)	7 (8.4)	10 (13.9)	44 (28.8)			
	College or above	14 (4.5)	2 (2.4)	2 (2.8)	10 (6.5)			
Smoking	No	260 (84.4)	70 (84.3)	57 (79.2)	133 (86.9)	2.242	0.326	chi-square
	Yes	48 (15.6)	13 (15.7)	15 (20.8)	20 (13.1)			
Alcohol use	No	248 (80.5)	64 (77.1)	57 (79.2)	127 (83.0)	1.303	0.521	chi-square
	Yes	60 (19.5)	19 (22.9)	15 (20.8)	26 (17.0)			
ADL category	Independent	184 (59.7)	39 (47.0)	45 (62.5)	100 (65.4)	9.345	0.009	H
	Mild dependence	107 (34.7)	35 (42.2)	24 (33.3)	48 (31.4)			
	Moderate dependence	9 (2.9)	5 (6.0)	2 (2.8)	2 (1.3)			
	Severe dependence	8 (2.6)	4 (4.8)	1 (1.4)	3 (2.0)			
Nutritional risk	No	234 (76.0)	65 (78.3)	55 (76.4)	114 (74.5)	0.435	0.804	chi-square
	Yes	74 (24.0)	18 (21.7)	17 (23.6)	39 (25.5)			
Pain on postoperative day 3	No pain	271 (88.0)	70 (84.3)	65 (90.3)	136 (88.9)	8.318	0.216	chi-square
	Mild pain	33 (10.7)	12 (14.5)	5 (6.9)	16 (10.5)			
	Moderate pain	3 (1.0)	0 (0.0)	2 (2.8)	1 (0.7)			
	Severe pain	1 (0.3)	1 (1.2)	0 (0.0)	0 (0.0)			
Pain on postoperative day 7	No pain	280 (90.9)	73 (88.0)	68 (94.4)	139 (90.8)	2.270	0.893	chi-square
	Mild pain	7 (2.3)	3 (3.6)	1 (1.4)	3 (2.0)			
	Moderate pain	15 (4.9)	5 (6.0)	2 (2.8)	8 (5.2)			
	Severe pain	6 (1.9)	2 (2.4)	1 (1.4)	3 (2.0)			
Multimorbidity	No	100 (32.5)	26 (31.3)	21 (29.2)	53 (34.6)	0.737	0.692	chi-square
	Yes	208 (67.5)	57 (68.7)	51 (70.8)	100 (65.4)			
Employment status	Employed	104 (33.8)	24 (28.9)	23 (31.9)	57 (37.3)	1.813	0.404	chi-square
	Not employed	204 (66.2)	59 (71.1)	49 (68.1)	96 (62.7)			
Place of residence	Urban	120 (39.0)	30 (36.1)	30 (41.7)	60 (39.2)	1.006	0.909	chi-square
	Town	80 (26.0)	24 (28.9)	16 (22.2)	40 (26.1)			
	Rural	108 (35.1)	29 (34.9)	26 (36.1)	53 (34.6)			
Monthly per-capita income, CNY	<1,000	47 (15.3)	36 (43.4)	3 (4.2)	8 (5.2)	86.337	<0.001	chi-square
	1,000-2,999	120 (39.0)	12 (14.5)	31 (43.1)	77 (50.3)			
	3,000-4,999	125 (40.6)	31 (37.3)	38 (52.8)	56 (36.6)			
	>=5,000	16 (5.2)	4 (4.8)	0 (0.0)	12 (7.8)			
Living arrangement	Not living alone	258 (83.8)	44 (53.0)	69 (95.8)	145 (94.8)	79.066	<0.001	chi-square
	Living alone	50 (16.2)	39 (47.0)	3 (4.2)	8 (5.2)			
Sleep quality	Very good	21 (6.8)	0 (0.0)	0 (0.0)	21 (13.7)	12.722	0.002	H
	Good	228 (74.0)	65 (78.3)	55 (76.4)	108 (70.6)			
	Poor	27 (8.8)	8 (9.6)	6 (8.3)	13 (8.5)			
	Very poor	32 (10.4)	10 (12.0)	11 (15.3)	11 (7.2)			

Data are n (%) unless otherwise specified. Continuous and ordered variables are presented as median (P25, P75) and compared with the Kruskal–Wallis H test. Nominal variables were compared with Pearson's chi-square test. BMI, body mass index; ADL, activities of daily living. Sparse cells in the pain and income tables warrant exact or Monte Carlo sensitivity analysis. Two-sided P<0.05 was considered statistically significant.

### 3.4 Factors associated with profile membership

The posterior-probability-weighted multinomial model produced the same pattern as the modal-assignment model. Compared with the high-performance profile, older age was associated with the low-performance profile (OR = 1.145, 95% CI 1.053–1.245, P = 0.002) and intermediate-performance profile (OR = 1.111, 95% CI 1.034–1.194, P = 0.004). Higher education was inversely associated with both profiles (low: OR = 0.285, 95% CI 0.181–0.447, P<0.001; intermediate: OR = 0.622, 95% CI 0.446–0.868, P = 0.005).

Greater ADL dependence (OR = 1.966, 95% CI 1.130–3.420, P = 0.017) and living alone (OR = 32.780, 95% CI 11.496–93.472, P<0.001) were associated with the low-performance profile, whereas each higher income category was associated with lower odds of membership in that profile (OR = 0.578, 95% CI 0.364–0.919, P = 0.020). Poorer sleep was associated with the intermediate-performance profile (OR = 1.583, 95% CI 1.057–2.371, P = 0.026). The extreme living-alone estimate and wide confidence interval indicate sparse-data inflation (**Table 1**).

## 4 Discussion

The principal finding is a severity gradient in day-7 MMSE performance, not evidence of postoperative decline or three distinct neurocognitive diagnoses. All five trajectories were similarly ordered, inter-domain Spearman correlations ranged from 0.578 to 0.771, and the first principal component explained 80.7% of standardized variance and correlated 0.998 with MMSE total score (**Supplementary Table S2**). Thus, the LPA offers a descriptive stratification of the observed score distribution but limited evidence of incremental multidimensional structure beyond general cognitive performance. The combined 50.3% in the two lower profiles is not an incidence estimate.

The cross-sectional day-7 profiles may reflect preoperative reserve and socioeconomic position as much as perioperative exposures. Continuous preoperative MMSE scores were not retained, so change scores or reliable-change indices could not be calculated. Moreover, all patients underwent surgery under general anesthesia and there was no regional-anesthesia or non-surgical comparison group. The findings therefore cannot isolate effects of anesthesia from surgery, hospitalization, preexisting vulnerability, or measurement effects.

Older age and lower education were common correlates of both lower-performance profiles, but neither should be interpreted as a perioperative cause. Age may index reduced physiological reserve, whereas education is related to cognitive reserve, health literacy, and MMSE test performance (3, 4). The MMSE is education-sensitive, and medical-record exclusion of diagnosed impairment may not identify undiagnosed mild cognitive impairment or dementia. Consequently, lower education and lower baseline cognitive reserve could partly explain class separation. Clinically, these non-modifiable characteristics may support additional screening with education-sensitive, multidomain tools rather than deterministic labeling.

ADL dependence was associated with the low-performance profile and may identify patients requiring broader functional assessment. Cognitive and physical function share correlates including mobility limitation, vascular burden, inflammation, malnutrition, and reduced engagement. Because ADL and cognition were assessed on the same postoperative day, directionality is uncertain. Pairing cognitive screening with functional assessment may help identify rehabilitation needs, but intervention efficacy must be tested prospectively (23).

The low-performance profile was also a distinct socioeconomic stratum, with markedly higher proportions of living alone, low income, and low education. Social disadvantage can limit cognitive stimulation, nutrition, medication support, rehabilitation access, and follow-up, while living alone may reduce observation of early cognitive problems (6, 24). Conversely, education-related MMSE bias, lower lifelong cognitive reserve, residual confounding, and undiagnosed preoperative disease may generate or amplify these associations without perioperative injury. The living-alone OR was extreme and imprecise because 39 of 50 participants living alone were in the low-performance profile; it should be viewed as a sparse-data signal requiring replication, not as a stable causal effect.

Poorer sleep was associated with the intermediate-performance profile, but sleep and cognition were measured concurrently on day 7. Sleep disruption can impair attention and memory consolidation through circadian, neuroendocrine, and inflammatory pathways and may interact with pain, anxiety, noise, and nighttime care (5, 25, 26). Reverse causation is also possible. Sleep promotion is a plausible low-risk nursing strategy, but the present association cannot establish that it will change cognitive outcomes.

These findings support cautious, hypothesis-generating stratification rather than immediate profile-prescribed care. Repeated cognitive assessment, functional evaluation, social-support review, and sleep assessment may identify patients who need closer observation or discharge support. Any tiered intervention based on these profiles requires prospective validation using baseline change, broader neuropsychological measures, delirium ascertainment, and clinically relevant outcomes.

Several limitations are important. First, continuous preoperative MMSE scores were not retained, preventing analysis of baseline-to-day-7 change or reliable change; the same MMSE format was repeated without an alternate form or practice-effect correction, which may have increased day-7 scores. Second, the MMSE is a screening tool with ceiling and education effects and limited executive-function assessment. Record review may have missed undiagnosed mild cognitive impairment or dementia. Third, the absence of regional-anesthesia and non-surgical comparison groups prevents attribution to general anesthesia. Fourth, unmeasured delirium, surgery type and complexity, anesthesia duration and depth, hypotension, blood loss, medication exposure, inflammation, and complications may confound associations. Fifth, concurrent day-7 sleep, ADL, and cognitive assessments preclude temporal inference. Finally, the single-center sample, univariate covariate screening, ordinal linearity assumptions, small intermediate class, and sparse living-alone cells limit generalizability and precision, although posterior-probability-weighted sensitivity analysis reduced concern about classification uncertainty.

## 5 Conclusion

Day-7 MMSE scores in older adults after elective surgery formed low-, intermediate-, and high-performance profiles that primarily represented a general severity gradient. Older age and lower education were associated with both lower-performance profiles; ADL dependence, lower income, and living alone characterized the low-performance profile, while poorer sleep characterized the intermediate profile. These exploratory associations may guide future screening research, but they do not demonstrate cognitive decline, diagnosis, or an effect of general anesthesia. Longitudinal multicenter studies with retained baseline cognition, comparison groups, comprehensive neuropsychological assessment, and perioperative confounder measurement are needed before profile-specific nursing interventions are implemented.

## Data Availability

The original contributions presented in the study are included in the article/**Supplementary Material**. Further inquiries can be directed to the corresponding authors.
