# Supplementary material for: Latent profiles of day-7 cognitive performance and associated ecological factors in older adults after elective surgery: an observational study
Source: Front Psychiatry. 2026 Oct 28;17:1975739. doi: 10.3389/fpsyt.2026.1975739 (PMC13752165; doi:10.3389/fpsyt.2026.1975739)
Supplement: Supplementary file 1 [file Table1.docx]

**Supplementary Table S1. Observed domain means, 95% confidence intervals, and standardized means by profile**

| **Profile** | **Domain** | **Mean (95% CI)** | **Standardized mean, z** |
| --- | --- | --- | --- |
| Low performance | Orientation | 6.518 (6.386–6.650) | -1.391 |
|  | Immediate registration/memory | 1.145 (1.028–1.262) | -1.258 |
|  | Attention and calculation | 2.422 (2.300–2.543) | -1.268 |
|  | Delayed recall | 0.819 (0.723–0.915) | -1.363 |
|  | Language and visuoconstruction | 4.988 (4.840–5.136) | -1.542 |
| Intermediate performance | Orientation | 8.361 (8.220–8.503) | -0.026 |
|  | Immediate registration/memory | 2.083 (1.969–2.198) | -0.144 |
|  | Attention and calculation | 3.569 (3.415–3.724) | -0.235 |
|  | Delayed recall | 2.014 (1.908–2.120) | -0.096 |
|  | Language and visuoconstruction | 8.458 (8.314–8.603) | 0.514 |
| High performance | Orientation | 9.431 (9.345–9.518) | 0.767 |
|  | Immediate registration/memory | 2.837 (2.778–2.895) | 0.750 |
|  | Attention and calculation | 4.719 (4.647–4.790) | 0.799 |
|  | Delayed recall | 2.843 (2.785–2.901) | 0.784 |
|  | Language and visuoconstruction | 8.595 (8.517–8.673) | 0.595 |

Standardized means use the full-sample mean and standard deviation for each domain. Confidence intervals are descriptive normal-approximation intervals based on modal class assignment.

**Supplementary Table S2. Spearman correlations among postoperative day-7 MMSE domains**

| **Domain** | **Orientation** | **Registration/memory** | **Attention/calculation** | **Recall** | **Language/visuoconstruction** |
| --- | --- | --- | --- | --- | --- |
| Orientation | 1.000 | 0.729 | 0.730 | 0.771 | 0.653 |
| Immediate registration/memory | 0.729 | 1.000 | 0.729 | 0.738 | 0.619 |
| Attention and calculation | 0.730 | 0.729 | 1.000 | 0.751 | 0.578 |
| Delayed recall | 0.771 | 0.738 | 0.751 | 1.000 | 0.638 |
| Language and visuoconstruction | 0.653 | 0.619 | 0.578 | 0.638 | 1.000 |

The first principal component explained 80.7% of variance in standardized domain scores and correlated r=0.998 with the MMSE total score, supporting a predominantly general severity dimension.
